# Factors Influencing Adherence to Auto-CPAP: An Observational Monocentric Study Comparing Patients With and Without Cardiovascular Diseases

**DOI:** 10.3389/fneur.2019.00801

**Published:** 2019-08-02

**Authors:** Ahmad Nsair, David Hupin, Stéphanie Chomette, Jean Claude Barthélémy, Frédéric Roche

**Affiliations:** ^1^Service de Physiologie Clinique et de l'Exercice, CHU de Saint-Etienne, Saint-Étienne, France; ^2^EA SNA EPIS 4607, Université de Lyon, Université Jean Monnet, Saint-Étienne, France

**Keywords:** CPAP (continuous positive airway pressure), cardiovascular disease, sleep apnea hypopnea syndrome, treatment adaptation, adherence - compliance - persistence, OSA (obstructive sleep apnea)

## Abstract

**Introduction:** Obstructive sleep apnea/hypopnea (OSAH) affects more than 15% of the general population and increases the occurrence of cardiovascular (CV) and metabolic events. Continuous positive airway pressure (CPAP) treatment is currently the gold standard treatment of OSAH and could prevent the occurrence of such events. However, long-term adherence to CPAP is a problem where a significant rate stop device treatment use. OSAH patients suffering CV disease could be less compliant due to less diurnal symptoms.

**Methods:** We performed a prospective study of 408 non-CV or CV disease patients suffering severe OSAH syndrome and followed them during the first 5 months as well as a mean of 3 years of CPAP treatment use.

**Results:** We demonstrated that in adult OSAH patients that two variables were associated with a low compliance (<5 h/night): age <60 y and lower maximal positive airway pressure level used. There was no significant impact of the presence of CV disease on compliance of 5 months. After 3 years of CPAP, age <60 y as well as diabetes were independent factors of low compliance. There was no significant association between gender, mask types, 90th centile positive airway pressure level, apnea/hypopnea index and short- or long-term compliance in our population.

**Conclusions:** We did not find lower compliance of CPAP in CV OSAH patients. Most of our population (68–73%) demonstrated an optimal night treatment duration at 3 years of follow-up, allowing a reduction of CV occurrence or recurrence. We hypothesize that an early and short education of OSAH as we routinely proposed could allow a significant increase in the optimal observance of CPAP in at-risk populations.

## Introduction

Obstructive sleep apnea/hypopnea (OSAH) has deleterious effects on human health, such as daytime somnolence, fatigue, irritability (1), high cardiovascular (CV) risks (2), and cognitive dysfunction and depression (3). Continuous positive airway pressure (CPAP) therapy is an effective treatment for OSAH which has given the best outcomes until now (4, 5). Therefore, it is important to investigate how to improve adherence and knowledge of CPAP treatment.

Several studies have shown that CPAP treatment depends on the patient, technological device factors, side effects, and psychological and social factors. Other factors like apnea/hypopnea index (AHI) and associated chronic disease (6) could play a role and also influence the level of compliance. A linear relationship between duration of CPAP use and outcome has been demonstrated with at least 5 h/night (7). Positive effect after 3 months of CPAP use for subjective sleepiness (Epworth sleepiness scale; ESS) (8), oxyhemoglobin desaturation index (9), heart rate and pulmonary artery pressure (10), and cognitive performance such as memory process was eight times superior for 6 h/night of use compared to ≤ 2 h/night (11, 12).

Smith et al. showed that with knowledge tests, such as the Apnea Knowledge test and Apnea Beliefs Scale (13), we could improve patients' knowledge about OSAH and at the same time improve treatment compliance and adherence to CPAP treatment, which increases the duration of CPAP use by the patients. Motivational enhancement can increase the perception of the positive aspects of CPAP (14). Psychological variables could also influence the adherence to treatment by CPAP. One of these important factors is that the patient needs to believe in his/her illness and its treatment efficiency (15). The level of involvement and education by healthcare professionals at the time of initiation of treatment can be a factor affecting adherence to CPAP treatment (16).

It has been estimated that 15–30% of patients do not accept CPAP treatment from the outset, i.e., before or during their titration study (17, 18). This early pattern of CPAP use is critical for determining continued patterns of use (19). Out of those who do initially accept CPAP treatment, 25–50% of patients fail to adhere optimally (20). In the long-term, up to 25% of patients stop using CPAP treatment by the third year (21).

The purpose of this prospective longitudinal study was to investigate factors and cofactors, which could modify (1) the first 5 months and (2) the first 3 years of compliance. We targeted a subpopulation with CV problems since it has been suggested that this population should be less symptomatic before CPAP treatment and therefore possibly less observant in the short- or long-term (22, 23).

## Methods

### Participants

Consecutive adult patients (*n* = 423) between 18 and 85 years old with or without CV diseases and OSAH were included in our study. All these participants were included consecutively in a 5-month follow-up study based on continuous monitoring by their medical service provider and their consultations at the VISAS Center of Saint Etienne University Hospital (France).

The level of education of patients was obtained by the number of years of institutional study (including elementary school). In France, more than 12 years of studies correspond to a level of education beyond the baccalaureate (higher education). Furthermore, marital status was included to determine whether patients lived alone in their home or had a spouse or a partner.

Inclusion criteria included patients with severe OSAH diagnosis by ambulatory polygraphy or ambulatory polysomnography (AHI>30/h for polygraphy or AHI>15/h associated with sleep fragmentation using polysomnography) and needed CPAP therapy. Exclusion criteria were contraindication or refusal of CPAP for home treatment. Patients with mixed apnea/hypopnea syndrome, central sleep apnea or systolic heart failure (LVEF<45% and/or symptoms of heart failure) were also excluded from the study. Hypothesizing a size effect of 0.2 for a power of 90% at a *p* < 0.05, an alpha power of analysis showed that a sample size of 120 patients per group is sufficient.

**Figure 1:**
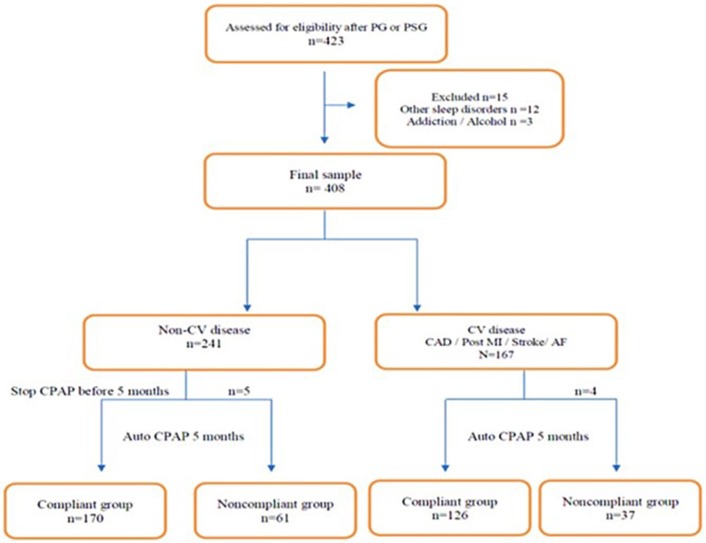
Flowchart of the population understudied according to the presence or the absence of a diagnosed cardiovascular disease at the entry in the study.

Figure 1 shows, together with compliance data, the number of patients lost during 5 months as well as during 3 years to follow-up in each group. Fifteen patients were excluded (12 patients with other sleep disorders and 3 patients with addiction and/or excessive alcohol consumption). The final group included 408 patients, most of them males (*n* = 219) categorized into two groups: non-CV (*n* = 236) and CV (*n* = 163) diseases. Five patients from the non-CV group stopped their CPAP treatment before 5 months as well as 4 patients from the CV group. The population of apneic patients with CV disease differed in that they were older, less obese, and had a higher proportion of men. On the other hand, the degree of daytime sleepiness and the prevalence of classical CV risk factors were not different between the two subpopulations (Table 1).

**Table 1:** Baseline characteristics of the OSAH population included in the study according to the presence of a recognized CV disease (coronary artery disease, post myocardial-infarction, stroke, atrial fibrillation).

**Variables**	**Non-CV OSAH patients *N* = 231**	**CV OSAH patients *N* = 163**	***P*-value**
**CATEGORY**			
Alone at home (%)	19.5	20.2	ns
Female (%)	33.7	15.3	<0.0001
PG (%)	54.1	61.3	ns
Auto-CPAP titration (%)	96.5	90.2	ns
Active smoker (%)	32.5	36.8	ns
History of hypertension (%)	38.5	36.2	ns
History of diabetes (%)	20.0	25.8	ns
**CONTINUOUS**			
Age (y)	58.9 ± 11.6	64.3 ± 11.3	<0.0001
Educational status (y)	11.1 ± 3.9	10.5 ± 4.0	ns
BMI (kg/m^2^)	31.2 ± 5.9	29.7 ± 4.4	<0.01
ESS score	9.9 ± 3.6	10.1 ± 3.4	ns
AHI (/h)	42.0 ± 7.5	44.2 ± 15.5	ns
ODI (/h)	37.4 ± 19.0	34.5 ± 17.1	ns

*PG, polygraphic recording; CPAP, continuous positive airway pressure; CV, cardiovascular disease; BMI, body mass index; ESS, Epworth somnolence scale; AHI, apnea plus hypopnea index; ODI, oxyhemoglobin desaturation index; OSAH, obstructive sleep apnea/hypopnea*.

The follow-up was then carried out up to 3 years after the initiation of the CPAP treatment (minimum 2 years and 4 months and maximum 5 years and 4 months) for the whole population studied.

Over an average follow-up period of 2.9 ± 0.8 years, we were able to record the occurrence of 12 deaths (mainly by CV events, *n* = 7). There were 6 deaths in each subgroup (CV or non-CV patients). Patients who died were not less observant than the rest of the study population but were statistically older (*p* < 0.02) when CPAP treatment had been initiated. We have not lost sight of our study. For the population still alive at the end of the follow-up (February 2019), we noted 49 stopped CPAP treatment (34 patients in the non-CV group vs. 26 patients in the CV group: *p* = 0.7).

The local Ethics Committee approved the study (CPP Sud Est 1, Saint Etienne, France: study ID 1508146; 2015-AO1635-44). The National Committee for Information and Liberty (CNIL) also gave consent for data collection. All subjects gave their informed and written consent prior to participation in the study.

### Auto-CPAP Adherence

All participants received initially an autotitration CPAP treatment device that contained a microchip (SmartCard^TM^) for remote monitoring. The compliance data presented represent an average duration of daily use of the device over the last 3 months of use. The memory of the CPAP machines was queried for each patient. Follow-up data were also collected for ease of use, mask leakage level, residual apnea/hypopnea index. When stopping treatment, the reason for this therapeutic stop was confirmed for each patient and validated by the doctor in charge of the patient. The type of mask used and the pressure settings were recorded. Optimal CPAP use was defined by mean h/night (<5 h/night or ≥5 h/night). Three types of masks were used: nasal, full face and pillows. Most of the CPAP machines were used with a humidifier (78%). Average hours of CPAP use at 5 months of treatment were used as outcome measures, since this corresponded to CPAP prescription renewal. After 5 months and after 3 years of treatment by CPAP, we observed a relatively high rate of compliance in CV patients as well as in non-CV subjects (Figure 1).

### Clinical Assessment

The clinical evaluation of patients was assessed by a standard medical consultation at 5 months for their prescription renewal or before if there was a medical need as well as at 3 years, which included general symptoms, sleeps symptoms, blood pressure and weight measurements, and subjective time and comfort of using CPAP, including the side effects. The monitoring also included evaluation of mean efficient positive airway pressure (PAP), and adjustment of maximal and minimal PAP.

Subjective sleepiness was assessed with a valid ESS administered initially (while the diagnosis of OSAH was done) and at 5 months of treatment. The ESS is an 8-item, self-administered questionnaire that measures subjective daytime sleepiness by assessing the self-reported likelihood of falling asleep in various settings (24, 25).

### Statistical Analysis

Descriptive statistics were generated for all continuous variables to describe the study population. Patients with incomplete data were not included in the study. Potential covariates included age, marital status, educational status, body mass index (BMI; kg/m^2^), AHI, CPAP pressure, ESS, and type of mask. We reduced the number of covariates in order to increase the degrees of freedom in the analyses. Patients were categorized into one of two groups: non-observant (<5 h/night) or observant (≥ 5 h/night). Such a categorization was done at 5 months as well as at 3 years of CPAP treatment. Odds ratios were calculated to assess the risk of adherence or non-adherence to CPAP therapy. Logistic regression analysis was then realized in order to determine factors associated with short-term as well as long-term adherence to treatment. Such logistic regression analysis was also done separately in CV OSAH and nonCV OSAH subpopulations. Data were analyzed by using Statview 5.0 (SAS Institute Inc., Stat Corp., College Station, TX, USA).

## Results

### Short-Term Follow-Up (5 Months)

**Figure 2:**
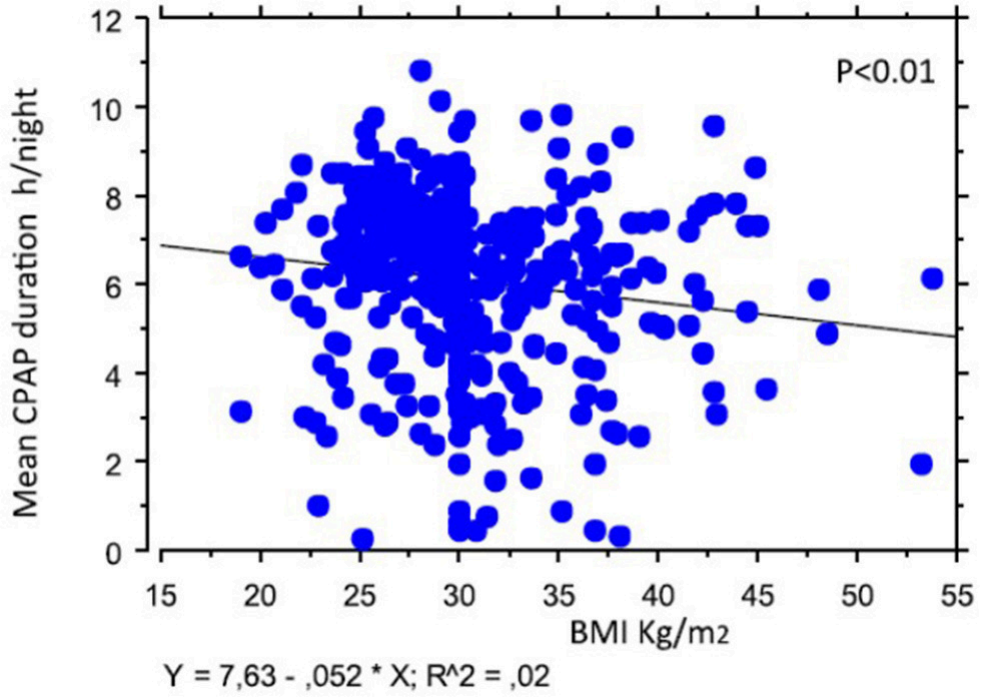
Linear regression analysis testing the relationship between mean nocturnal CPAP duration and body mass index at the inclusion in the study.

In Table 1, we depict the main characteristics of our population at point of inclusion. Baseline ESS score as well as AHI or ODI were not significantly different in the two subpopulations of apneic patients. Univariate linear regression analysis showed that mean time duration of CPAP treatment (continuous variable) was negatively correlated with BMI (*R* = 0.16; *R*^2^ = 0.03; *p* < 0.01) (Figure 2). Patients with higher BMI were less observant to CPAP treatment. There was no significant association between mask types used or need of mask switch, mean PAP level (auto-CPAP patients only in the analysis), use of humidifier, and 5 months compliance in our selected population (Table 2). These results were true in CV patients as well as in non-CV patients. Therefore, we could observe a favorable impact of CPAP treatment on daytime hypersomnolence in both non-CV and CV OSAH patients.

**Table 2:** Five months and 3 years characteristics of the OSAH population studied according to the presence at the inclusion of a recognized cardiovascular disease [coronary artery disease, post myocardial–myocardial infarction (*n* = 102), stroke (*n* = 43), atrial fibrillation (*n* = 18)].

**Variables**	**Non-CV OSAH patients**	**CV OSAH patients**	***P*-value**
**5 months category**	***N*** **=** **231**	***N*** **=** **163**	
Short-term observance >5 h (%)	73.6	77.3	ns
Humidifier (%)	62.4	55.6	ns
Mask full face (%)	19.9	26.4	ns
Nasal (%)	72.4	67.5	ns
Nasal pillows (%)	7.7	6.1	ns
Mask type switch (%)	23.0	20.2	ns
Excessive leaks (%)	31.5	34.9	ns
Comfort, satisfaction (%)	88.3	89.6	ns
**5 months continuous**			
Residual ESS score	4.9 ± 3.7	5.1 ± 3.4	ns
Mean observance (h/n)	6.1 ± 1.9	6.0 ± 2.0	ns
Residual AHI (/h)	2.3 ± 2.4	3.2 ± 3.3	<0.05
90th centile auto-PAP	11.1 ± 2.3	11.0 ± 2.0	ns
Max auto-PAP	14.0 ± 1.7	13.1 ± 1.9	<0.01
Min auto-PAP	4.4 ± 0.7	4.4 ± 0.8	ns
**3 years category**	***N*** **=** **225**	***N*** **=** **157**	
Long-term observance >5 h (%)	67.8	72.6	ns
Excessive leaks (%)	32.4	30.3	ns
Comfort, satisfaction (%)	88.4	89.8	ns
**3 years continuous**			
Residual ESS score	5.3 ± 3.4	5.5 ± 3.7	ns
Mean observance (h/n)	6.0 ± 2.2	6.3 ± 2.0	ns
Residual AHI (/h)	2.2 ± 2.2	3.4 ± 4.1	<0.005
90th centile auto-PAP	11.0 ± 2.0	10.7 ± 2.2	ns
Max auto-PAP	14.0 ± 1.4	13.2 ± 1.4	<0.01
Min auto-PAP	4.4 ± 0.6	4.4 ± 0.5	ns

*PAP, positive airway pressure; ESS, Epworth somnolence scale; AHI, apnea plus hypopnea index*.

We found that in the 5 months mean observance duration, the 90th centile of PAP or minimal PAP were not statistically different between the two subpopulations, but residual AHI was slightly higher and maximal PAP lower in CV patients (Table 2).

A logistic regression model was built to evaluate the variables independently associated with CPAP use at >5 h/night in the whole population included in the study. In this model, after adjustment for gender, BMI and pre-treatment AHI, we found that age (60–70 or >70 years) and maximal PAP were the only two significant variables predictive of a good compliance (Table 3).

**Table 3:** Logistic regression analysis realized on the global OSAH population (CV plus non-CV patients) to determine the best model of independent predictors of an optimal CPAP observance (more than 5 h/night at 5 months of treatment) after full adjustment.

**Variables in the model (all patients)**	**OR**	**95% CI**	***P*-value**
Ref <60 y	1	-	
Age 60–70 y	2.63	1.52–4.70	<0.01
Age >70 y	2.81	1.40–5.41	<0.01
Max. auto-CPAP (+1 cm H_2_O)	1.22	1.05–1.37	<0.01

*CPAP, continuous positive airway pressure, CV, cardiovascular, OSAH, obstructive sleep apnea/hypopnea*.

According to subgroup analysis, the same variables were found in the model as significant independent predictors of a good compliance in the non-CV OSAH patients. However, none of those variables reached a significant level in the CV OSAH group, probably due to a smaller sample size (Table 4).

**Table 4:** Logistic regression analysis realized on non-CV subpopulation (upper panel) and on CV OSAH subpopulation in order to determine independent predictors of an optimal CPAP observance (at least 5 h/night at 5 months of treatment) after full adjustment.

	**OR**	**95% CI**	***P*-value**
**VARIABLES IN THE MODEL (NON-CV PATIENTS)**			
Ref <60 y	1	-	
Age 60–70 y	2.67	1.28–5.47	<0.01
Age >70 y	2.89	1.13–7.37	<0.03
Max. auto-CPAP (+1 cm H_2_O)	1.22	1.03–1.46	<0.03
**VARIABLES IN THE MODEL (CV PATIENTS)**			
Ref <60 y	1	-	
Age 60–70 y	2.45	0.93–6.60	0.07
Age >70 y	2.61	0.93–6.67	0.07
Max. auto-CPAP (+1 cm H_2_O)	1.19	0.96–1.46	0.11

*CPAP, continuous positive airway pressure; CV, cardiovascular; OSAH, obstructive sleep apnea/hypopnea*.

### Long-Term Follow-Up (3 Years)

The factors associated with the maintenance of a 3-year CPAP treatment use in statistical analysis testing were the following: absence of diabetes (*p* < 0.002), older age (*p* = 0.03), higher effective pressure in the 90th percentile (11.1 ± 2.1 vs. 10.3 ± 2.9 cm H_2_O) measured after the first 5 months of treatment (*p* < 0.001), and a longer CPAP use (6.25 ± 1.7 vs. 4.46 ± 2.5 h/night) after the first 5 months of treatment (*p* < 0.001).

In subgroup analysis, these same previously described parameters associated with long-stay compliance were found in the group of non-CV patients; whereas in the CV group, only the longer initial 5-month adherence was associated with long-term adherence (*p* < 0.01).

The initial AHI, the index of residual respiratory events at 5 months or at 3 years, the initial ESS score, the evolution of this score (ESS) at 5 months and at 3 years, initial BMI, gender, marital status, and educational level were not associated in our whole population at 3 years and whether patients had CV status (CV or non-CV).

Comparing the average value of the duration of CPAP use at 3 years in patients who kept their active treatment, there was no difference in the duration of use between the subgroups (CV or non-CV): 6.44 ± 1.97 vs. 6.19 ± 2.27 h/night, respectively. On the other hand, CV patients who spontaneously discontinued CPAP during follow-up had a better average compliance than non-CV patients during the last 6 months prior to discontinuation (4.62 ± 2.65 vs. 2.27 ± 2.03 h/night, *p* < 0.04).

It should be noted that the residual AHI in patients still under CPAP at 3 years was low regardless of the treatment method used (2.9 ± 3.1 events/h). On the other hand, the treated CV patients kept a discreetly higher residual AHI (3.4 ± 4.1 vs. 2.2 ± 2.2 events/h, *p* < 0.005). If we looked at the target observance of 5 h (or more) per night in the population, including patients who stopped CPAP treatment, we did not find a difference in the proportion of patients who were observant in the two subgroups: 72.6% in CV patients 67.8%in non-CV patients.

Tables 5, 6 show with multiple logistic regression analysis the factors found to be independently associated with an adherence of >5 h in the whole population and in each of the two subgroups of patients who were still under CPAP at the end of the 3-year follow-up.

**Table 5:** Logistic regression analysis realized on the global OSAH population (CV plus non-CV patients) to determine the best model of independent predictors of an optimal CPAP observance (more than 5 h/night at 3 years of treatment) after full adjustment.

**Variables in the model (all patients)**	**OR**	**95% CI**	***P*-value**
Ref <60 y	1	-	
Age 60–70 y	1.54	0.92–2.58	0.10
Age >70 y	1.32	0.76–2.31	0.31
Diabetes (Yes)	0.57	0.34–0.94	<0.03

*CPAP, continuous positive airway pressure; CV, cardiovascular; OSAH, obstructive sleep apnea/hypopnea*.

**Table 6:** Logistic regression analysis realized on non-CV subpopulation and on CV OSAH subpopulation in order to determine independent predictors of an optimal CPAP observance (at least 5 h/night at 3 years of treatment) after full adjustment.

	**OR**	**95% CI**	***P*-value**
**VARIABLES IN THE MODEL (NON-CV PATIENTS)**			
Ref <60 y	1	-	
Age 60–70 y	1.89	1.02–3.49	<0.05
Age >70 y	1.65	0.74–3.72	0.22
Diabetes (Yes)	0.36	0.19–0.71	<0.01
**VARIABLES IN THE MODEL (CV PATIENTS)**			
Ref <60 y	1	-	
Age 60–70 y	1.06	0.43–2.61	0.89
Age >70 y	0.85	0.36–2.04	0.72
Diabetes (Yes)	1.19	0.44–2.21	0.99

*CPAP, continuous positive airway pressure; CV, cardiovascular; OSAH, obstructive sleep apnea/hypopnea*.

These data confirmed the results observed earlier in the 5-month follow-up for the those aged >60 y, but it also found the presence of diabetes as a factor limiting the maintenance of adherence at 3 years. Maximal PAP was no longer found in this analysis. Finally, in the subjective factors (patient interrogation) of the observance of more than 5 h to 3 years one also finds the notion of a good comfort of the patient (visual analogical scale >8/10) (*p* < 0.001).

## Discussion

The issues of health risks of OSAH make the demand of CPAP treatment important.

The aim of our study was to investigate the possibility to improve adherence to CPAP therapy in a CV population suffering severe OSAH (AHI>30/h).

In our study, we did not observe a significant difference in the rate of short-term or long-term adherence to CPAP therapy between our two subgroups (non-CV and CV patients). In addition, patients showed quite good CPAP adherence after 5 months of treatment.

Our results showed the possibility of obtaining good compliance with CPAP in a population of patients with CV pathology. An observance of more than 5 h was found in more than 70% of our population after 3 years of follow-up. This highlights the value of working with patients to improve adherence in the first few months of treatment and perhaps even the first few days. The factors of “good compliance” are represented by the age of >60 years, which is a factor found on initial compliance as well as on late compliance. On initial compliance, the high-pressure requirements are predictors of good adherence; whereas at the end of 3 years, the presence of diabetes is a factor that is detrimental to the maintenance of long lasting nocturnal ventilatory treatment.

As already demonstrated by others, the minimum use of CPAP efficacy treatment of ≥5 h/night for CV patients (22) appears important as a secondary CV prevention goal. An early education was associated with a patient's better knowledge about OSAH as a health problem and its therapy; this can improve adherence to CPAP treatment particularly for CV patients (13–15) who probably need a higher duration of CPAP use (26). Previous studies identified patients with CV disorders using CPAP therapy <3.5 h/night did not prevent CV events (22, 23). Our target was then to reach a duration of 5 h/night or more of CPAP use in our study. The compliance to CPAP therapy will be paramount to achieve their beneficial effects in our population (27). Motivational enhancement can play an important role in increasing CPAP adherence until 99.0 min/night compared to a control group that can reach a level of 5 h/night (14, 28). Socioeconomic and cognitive factors may also be associated with CPAP adherence (29). Comorbidities such as chronic obstructive pulmonary disease, asthma and rhinitis, and obesity could also have a negative impact on CPAP treatment.

In our adult population, we identified some variables that can be potentially associated with low adherence, such as age, higher BMI, and lower maximal CPAP pressure level. CV disorders, hypertension and diabetes have not been found to be predictors of lower adherence. Maximal CPAP level is routinely known as a factor of lower adherence to CPAP treatment (fixed PAP) because of uncomforting and more air leakage generation. Our result could appear somewhat surprising. Nevertheless, there was no significant association between mask types used, mean PAP level, AHI baseline value, or humidifier use and observance in our patients.

There is a not negligible factor that we eliminated from our study but frequently encountered in other already published studies, which is that the patients did not know the optimal duration we had chosen in the study. The medical and paramedic staff encouraged them to use their CPAP treatment as long as they could tolerate according to their night habits (usual sleep duration). Then we removed a factor that can increase the compliance for CPAP treatment compared to patients who knew that they were enrolled in a follow-up study (30). The technicians of their medical service providers gave the pick-up of our outcomes during their routine visits at home and during in-hospital stays (which can also increase the compliance to CPAP treatment). We think that this way, we could be close to the real CPAP treatment without any external intervention or direct contact with the patients, which can disrupt their real management.

There are some limits in our study, in that the number of included patients were different in the two subgroups (*n* = 236 non-CV and *n* = 163 CV). Such difference in sample sizes could explain the discrepancy of our logistic regression analysis according to the presence or not of a CV disease. In France, the management of OSAH and its treatment by CPAP maybe more efficient or better followed due to an easier access to a health protection system, more speedy diagnosis procedure, as well as CPAP device installation. We can highlight the real implication of medical providers in the explanation of CPAP therapy procedure and an element of comfort according to home interventions according to patient needs. There were few factors associated with a good compliance in our population. The elderly could be first interpreted as a factor limiting CPAP duration. This was not the case in our selected population that showed us an opposite result. The elderly are probably more anxious by potential CV events and then could be more observant compared to young adults. There is no consensus if we should consider OSAH as a single disease with different phenotypes with or without excessive daytime somnolence (31), or if there are different diseases with different genetic/epigenetic determinants, pathogenic mechanisms, prognosis, and treatment.

Thus we propose to realize in the future a randomized cohort study, which will be based on the impact of an early in-hospital information and education program for patients with recently diagnosed OSAH (in the first 2 weeks after OSAH diagnosis and one telephone call per month for 5 months). The aim of this future study will be to better identify the variables that can also positively influence adherence to CPAP therapy (32). We believe that the advice of CPAP therapy during several months could also help patients to better integrate risks of untreated OSAH and to balance the benefit of treatment.

## Conclusion

The effectiveness in terms of CV prevention of the treatment of OSAH syndrome is based, as for all risk factors, on adherence to therapy (in this case the mean nocturnal CPAP use). A threshold of 5 h/night has to be proposed in this context and it is not easy to obtain systematically. Therefore, it could appear essential to better recognize favorable or deleterious factors in order to optimize observance of CPAP treatment. Moreover, in secondary prevention, it is suggested that “CV” apneic patients would be less observant because they have less symptoms during daytime as was found in the SAVE study. We aimed to determine on a prospective cohort observational study the variable associated with a 5-month good observance in two populations of patients: with a history of CV disease (acute coronary syndrome, atrial fibrillation, stroke) or without such a CV event. We did not find a difference in terms of adherence rate between the two groups at 5 months and 3 years of follow up. The population studied presented two predictors of short-term good observance: older age (over 60 or over 70 years) and high maximum PAP treatment in the auto-CPAP group. Age appears still a factor of good observance at 3 years but diabetes also limits independently such a long-term adherence. Severity of the symptom of sleep related to breathing disorder, history of hypertension, AHI, marital status, educational level and gender do not seem to play a significant role on compliance at 5 months or at 3 years of treatment. Thus, it could be time now to propose a reinforcement of knowledge of OSAH syndrome as well as recognition of the favorable impact of CPAP treatment more particularly in young patients (33). Now, patient education and follow-up via telemedicine is used to improve adherence and could positively (or not) influence the healthcare provider in OASH following (34).

## Ethics Statement

All procedures performed in studies involving human participants were in accordance with the ethical standards of the institutional and/or national research committee and with the 1964 Helsinki declaration and its later amendments or comparable ethical standards.

## Informed Consent

Informed and written consent was obtained from all individual participants included in the study.

## Author Contributions

FR and AN involved in study design and drafting the manuscript. FR, DH, SC, and JB participated at inclusion and data acquisition. FR made statistical analysis. DH, SC, and JB proofreading the manuscript.

### Conflict of Interest Statement

All authors certify that they have no affiliations with or involvement in any organization or entity with any financial interest (such as honoraria; educational grants; participation in speakers' bureaus; membership, employment, consultancies, stock ownership, or other equity interest; and expert testimony or patent-licensing arrangements), or non-financial interest (such as personal or professional relationships, affiliations, knowledge, or beliefs) in the subject matter or materials discussed in this manuscript.
